# Preeclampsia as a Risk Factor of Postmenopausal Cardiovascular Disease: A Cross-Sectional Study

**DOI:** 10.3390/clinpract15070126

**Published:** 2025-07-02

**Authors:** Pasquale Palmiero, Pierpaolo Caretto, Francesca Amati, Marco Matteo Ciccone, Maria Maiello

**Affiliations:** 1 ASL Brindisi, Cardiology Equipe, District of Brindisi, 72100 Brindisi, Italy; mariamaiello@iciscu.org; 2 Medical School, University of Bari, 70122 Bari, Italy; 3 University Cardiology Unit, Interdisciplinary Department of Medicine, Polyclinic University Hospital, 70124 Bari, Italyfrancesca.amati@uniba.it (F.A.);

**Keywords:** preeclampsia, cardiovascular disease, postmenopausal, left ventricular hypertrophy, diastolic dysfunction

## Abstract

**Introduction:** Preeclampsia (PE) is a pregnancy-specific disorder characterized by hypertension and organ dysfunction, affecting 5–8% of pregnancies globally and increasing women’s long-term risk of cardiovascular disease (CVD). This study investigates the association between prior PE and cardiovascular health in postmenopausal women. **Methods:** A total of 108 postmenopausal women with a history of PE and 100 controls without PE were enrolled. Clinical data, blood pressure readings, and echocardiographic assessments were obtained. Statistical analysis was conducted using SPSS version 20.0. **Results:** Women with prior PE showed a higher prevalence of eccentric left ventricular hypertrophy (37% vs. 23%, *p* < 0.02) and diastolic dysfunction (51% vs. 39%, *p* < 0.003). Maternal history of hypertension was also more common in the PE group (55% vs. 26%, *p* < 0.003). Obesity was more frequent in the PE group, but did not reach statistical significance (*p* < 0.09). **Conclusions:** Prior PE was linked to an increased risk of postmenopausal cardiac abnormalities, including left ventricular hypertrophy and diastolic dysfunction. A maternal history of hypertension was also more common among women with prior PE, suggesting a familial connection; PE should be acknowledged as a significant predictor of long-term cardiovascular risk, requiring lifelong monitoring and preventive measures.

## 1. Introduction

Preeclampsia (PE) is a hypertensive disorder of pregnancy characterized by elevated blood pressure and multi-organ involvement, typically affecting the liver and kidneys. It typically develops after 20 weeks of gestation and may also occur postpartum. Globally, it affects 5–8% of pregnancies and is a major contributor to maternal and fetal morbidity and mortality. Although its exact pathogenesis is not fully elucidated, preeclampsia is believed to result from abnormal placental development, leading to inadequate remodeling of the spiral arteries. This causes widespread endothelial dysfunction, oxidative stress, and systemic inflammation. Risk factors include nulliparity, multiple gestation, pre-existing hypertension, diabetes, obesity, and autoimmune disorders.

Clinical manifestations range from asymptomatic hypertension detected during routine prenatal care to severe symptoms like proteinuria, headaches, visual changes, and abdominal pain. If unmanaged, preeclampsia can progress to life-threatening complications such as eclampsia, stroke, organ failure, and preterm birth. The only definitive treatment is delivery, though management often involves close surveillance, antihypertensive therapy, and low-dose aspirin prophylaxis during pregnancy for high-risk individuals.

Despite being classically defined as a pregnancy-related condition, there is increasing recognition of the long-term health implications of preeclampsia, particularly concerning cardiovascular health. Importantly, preeclampsia is now recognized not only as a pregnancy complication but also as a predictor of long-term cardiovascular disease (CVD) in women. Several studies have demonstrated that women with a history of PE are at increased risk of hypertension, ischemic heart disease, heart failure, and stroke later in life [1,2]. This increased risk is attributed to shared mechanisms such as endothelial dysfunction, chronic inflammation, and metabolic dysregulation [3,4].

The association between PE and subsequent cardiovascular morbidity is not merely coincidental. Rather, PE may serve as an early marker of an individual’s underlying vascular and metabolic susceptibility, unmasked by the physiological demands of pregnancy. Women with early-onset, recurrent, or severe preeclampsia, and those delivering preterm, face up to a twofold increased risk of developing CVD [2,5]. Moreover, preeclampsia often precedes or coexists with other CVD risk factors such as obesity, diabetes, and dyslipidemia [3,6]. Recognizing this, the American Heart Association includes PE in its cardiovascular risk guidelines [4].

The cardiovascular consequences of preeclampsia are not limited to the affected women. A maternal history of PE has also been linked to increased cardiovascular risk in offspring. Daughters of affected mothers are more likely to experience PE themselves and may inherit a predisposition to CVD, suggesting a genetic or epigenetic basis [5]. This intergenerational pattern implies that PE may have long-lasting consequences that transcend the immediate perinatal period and impact public health at a population level. PE falls under the spectrum of hypertensive disorders of pregnancy (HDPs), including gestational hypertension, chronic hypertension, and superimposed PE [6]. HDPs are a leading cause of perinatal complications and long-term cardiovascular issues. Postpartum monitoring is critical, especially for those with persistent hypertension. Women who develop PE should therefore not only be followed in the postpartum period but also be enrolled in long-term surveillance programs to mitigate the risk of future cardiovascular events. Notably, PE is a strong risk factor for developing chronic hypertension. Up to 50% of affected women develop sustained high blood pressure within 5 to 15 years [3,7]. The risk is heightened in cases of early-onset or severe PE and is driven by factors such as endothelial dysfunction [8], oxidative stress [9], inflammation, RAAS dysregulation [10], and genetic influences. These alterations can persist long after delivery and contribute to the development of a chronic hypertensive phenotype. These chronic changes may evolve into hypertensive cardiomyopathy, characterized by changes in left ventricular mass and function, often accompanied by left atrial remodeling and, eventually, diastolic dysfunction [11]. In such cases, early cardiac structural and functional abnormalities may be clinically silent for years before manifesting as overt heart failure or arrhythmias. Additionally, global pulse wave velocity (PWVg), a marker of aortic stiffness, has increased in women with a history of prior PE, highlighting early vascular aging [12]. These findings support the concept that preeclampsia may initiate or accelerate atherosclerotic and arteriosclerotic processes. Taken together, these findings emphasize the importance of considering preeclampsia as an early warning sign of future cardiovascular disease, warranting lifelong monitoring and prevention strategies. Obstetric history should therefore be an integral part of cardiovascular risk assessment, particularly in women presenting with hypertension or other risk factors later in life. Clinical guidelines increasingly support this integration, recommending routine cardiovascular screening for women with a history of PE, starting as early as the first postpartum year.

This study aims to determine whether preeclampsia during reproductive years is associated with increased cardiovascular disease risk due to hypertension in postmenopausal women. We also investigate whether maternal history of PE is common in menopausal women who had PE and whether such a history warrants targeted cardiovascular monitoring in their daughters. By examining these associations, we aim to contribute to a growing body of evidence supporting tailored prevention strategies that consider obstetric history as a critical component in women’s cardiovascular care.

## 2. Methods and Materials

We enrolled 108 consecutive postmenopausal women (aged 48–67 years) with a history of preeclampsia (PE) during their reproductive years, defined as new-onset hypertension during pregnancy associated with proteinuria, by the criteria of the International Society for the Study of Hypertension in Pregnancy (ISSHP). All participants were normotensive before pregnancy and experienced a return to normal blood pressure levels within three months postpartum. At the time of enrollment, all women had developed hypertension requiring pharmacological treatment only after the onset of menopause. Women with known chronic hypertension were excluded, along with chronic kidney disease, type 1 or type 2 diabetes mellitus, autoimmune disorders (e.g., systemic lupus erythematosus, antiphospholipid syndrome), and pregnancies conceived via assisted reproductive technologies. A control group (CG) of 100 consecutive postmenopausal women without a history of PE, referred to our clinic for menopausal symptoms by general practitioners, was also enrolled. Characteristics of the study population are in Table 1. Participants provided written informed consent. Baseline data included age, height, weight, BMI, duration of menopause, and personal and clinical history. Blood pressure (BP) was measured on the non-dominant arm using a calibrated sphygmomanometer, after at least 5 min of rest in the supine position, with the cuff covering at least 80% of the upper arm circumference. The mean of three consecutive measurements was recorded as the individual BP value. All participants underwent electrocardiographic and transthoracic echocardiographic (TTE) evaluation. Echocardiographic assessments were performed using an Epiq 7 ultrasound system (Philips Healthcare, Milan, Italy), equipped with a multifrequency transducer. Examinations were conducted with patients in the left lateral decubitus position, after a 10 min rest, with the examination table inclined at 30°. Echocardiographic parameters included septal/posterior wall thickness, LV diameters, ejection fraction, and fractional shortening. Diastolic function was assessed using a pulsed-wave Doppler at the mitral valve to measure peak early (E) and late (A) diastolic filling velocities and deceleration time. Tissue Doppler imaging was used to assess early diastolic (e′) and late diastolic (a′) velocities at the septal and lateral mitral annulus. The E/A, e′/a′, and average E/e′ ratios were calculated. Tricuspid regurgitation velocity (TRV) was estimated when feasible, and left atrial (LA) volume was calculated. Left ventricular mass (LVM) was determined using the Devereux formula and indexed to body surface area (BSA) to obtain the LVM index, with reference values based on the guidelines of the American Society of Echocardiography. Left ventricular diastolic dysfunction (LVDD) was diagnosed according to current recommendations for patients with preserved systolic function. When TRV was not measurable, a value <2.8 m/s was assumed. GLS was not assessed due to time limitations and its limited relevance in this population.

### Statistical Analysis

We performed using SPSS version 20.0 for Windows (SPSS Inc., Chicago, IL, USA). We checked normality for each variable and applied the Chi-square test for categorical variables; continuous variables were summarized as mean/SD (standard deviation). The odds ratio (OR) for MVP, its standard error, and 95% confidence interval (CI) were calculated. Results with *p* values less than 0.05 were considered statistically significant.

## 3. Results

**Figure 1:**
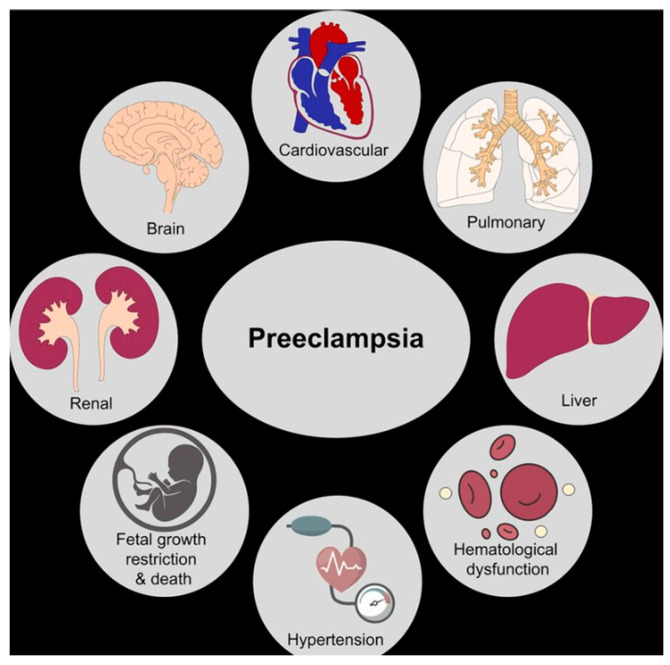
Multisystem impact of preeclampsia on maternal organs and cardiovascular function.

In our cohort, women with a history of preeclampsia (PE) exhibited a significantly higher prevalence of cardiovascular structural and functional abnormalities compared with those without such a history. Notably, eccentric left ventricular hypertrophy was observed in 40 women (37%) with a history of prior PE compared with 23 women (23%) in the control group. This difference reached statistical significance (*p* < 0.02), with a Chi-square value of 4.8 and an odds ratio (OR) of 1.9, indicating an almost two-fold increased risk in the PE group; similarly, left ventricular diastolic dysfunction, a key indicator of impaired myocardial relaxation and early heart failure risk, was significantly more prevalent among PE-affected women, affecting 56 individuals (51%) compared with 39 (39%) in the control population. This finding showed robust statistical significance (*p* < 0.003), with a Chi-square of 13.2 and an OR of 2.6, underlining a strong association between preeclampsia and subsequent diastolic dysfunction (Table 2 and Table 3 and Figure 1). Although obesity was more frequent among women with a history of PE (52 cases, 39%) than among controls (28 cases, 28%), the difference did not reach conventional levels of statistical significance (*p* < 0.09), with a Chi-square value of 2.7 and an OR of 1.6. Nonetheless, this trend supports the known metabolic implications of preeclampsia and suggests a possible contribution to long-term cardiovascular risk; however, the lack of statistical significance precludes definitive conclusions in the current cohort and indicates that larger studies are needed to confirm the association. A significantly greater proportion of women in the PE group also reported a maternal history of arterial hypertension (59 women, 55%) compared with controls (26 women, 26%). This association was statistically significant (*p* < 0.003), with a Chi-square value of 8.4 and an OR of 2.3, further supporting the hypothesis of a familial or genetic predisposition to hypertensive disorders of pregnancy and their cardiovascular sequelae. No significant differences were found between groups regarding the prevalence of left atrial enlargement, which affected 24 women (22%) with prior PE and 15 women (15%) in the control group (*p* < 0.1, Chi-square 1.8, OR 1.6), suggesting that this parameter may be less sensitive in differentiating long-term cardiac remodeling in this population (Table 2 and Table 3 and Figure 1).

## 4. Discussion

This study adds to the growing body of evidence linking preeclampsia (PE) with long-term cardiovascular risk in women. Moreover, it documents, through ultrasonographic methods, the morphological and functional changes occurring at the cardiac level in women with PE [13,14,15]. While the acute manifestations of PE during pregnancy are well-documented, our findings highlight the enduring cardiovascular impact of the condition, particularly as women transition into the postmenopausal period [16,17]. Specifically, our results demonstrate a significantly higher prevalence of eccentric left ventricular hypertrophy and diastolic dysfunction in postmenopausal women who experienced PE compared with those who did not. These findings suggest that PE is not merely a transient gestational complication but may serve as a sentinel event that unmasks or accelerates underlying cardiovascular vulnerability [18,19]. The cross-sectional nature of our study prevents definitive causal inference. However, the consistency of our results with the prior literature supports the plausibility of a lasting link between PE and adverse cardiac remodeling. Eccentric left ventricular hypertrophy (LVH), observed in 37% of the PE group compared with 23% of controls, is a form of cardiac remodeling often associated with volume overload and sustained hypertension. This is consistent with previous evidence indicating that chronic hypertension and volume overload contribute significantly to LVH development [20,21]. The presence of eccentric LVH in our cohort supports the notion that women with a history of PE are more susceptible to maladaptive cardiac changes later in life, likely mediated by the chronic hypertension that commonly emerges post menopause. Additionally, the persistence of hemodynamic and inflammatory stressors initially triggered by PE may promote ongoing cardiac remodeling even years after delivery. Moreover, previous studies confirm that the cardiovascular effects of PE persist long after delivery and may contribute to structural heart disease [22]. Even more striking is the increased prevalence of diastolic dysfunction (DD) among women with prior PE (51% vs. 39%). Diastolic dysfunction is a known precursor to heart failure with preserved ejection fraction (HFpEF), a condition increasingly recognized in older women and closely associated with hypertension, obesity, and metabolic disease [23]. The strong statistical association (*p* < 0.003, OR 2.6) found in our cohort suggests that the pathophysiological processes initiated during a PE-complicated pregnancy, such as endothelial dysfunction, oxidative stress, and vascular inflammation, may continue or resurface years later, contributing to myocardial stiffness and impaired relaxation [24]. The observation of such a significant difference in DD prevalence reinforces the need for early identification and management of women at risk, even decades after pregnancy. While obesity did not show statistically significant differences between groups, the observed trend (39% vs. 28%, *p* < 0.09) aligns with the metabolic syndrome profile frequently associated with both PE and later-life cardiovascular disease. Obesity itself may be both a contributor to and a consequence of cardiovascular remodeling, particularly when coupled with insulin resistance and chronic inflammation [25]. This bidirectional relationship underscores the complexity of the metabolic cardiovascular continuum that PE may initiate or exacerbate. The higher prevalence of obesity in the PE group may thus reflect a shared pathophysiological pathway linking gestational hypertensive disorders and postmenopausal cardiovascular risk [26]. One of the most novel and intriguing aspects of our study is the observation that women with a history of PE were more likely to have mothers who also suffered from hypertension (55% vs. 26%). This finding suggests a potential genetic or epigenetic component to the intergenerational transmission of hypertensive disorders [27]. The familial aggregation of hypertensive pathology could indicate the presence of inherited susceptibility loci, shared environmental influences, or epigenetic modifications induced in utero. Previous research has suggested that daughters of women with PE are at an increased risk of the condition themselves, and our data further supports this familial clustering of hypertensive pathology. Given the substantial overlap in risk factors and the emerging understanding of fetal programming, it may be appropriate to consider maternal history of PE or hypertension as a significant risk factor, warranting closer monitoring in pregnancy planning and management for daughters [28]. Preventive strategies in women with a maternal history of PE could include preconception counseling, more intensive monitoring during pregnancy, and long-term cardiovascular follow-up. Interestingly, no statistically significant difference was observed in the prevalence of left atrial (LA) enlargement between the two groups. While LA size is a useful surrogate for chronic diastolic burden, it may not be sufficiently sensitive to detect early or subclinical stages of cardiovascular remodeling in this population. Alternatively, it is possible that diastolic dysfunction in the PE group had not yet progressed to the point of inducing measurable atrial dilation [29]. It is also plausible that the echocardiographic criteria for LA enlargement may not adequately capture functional atrial impairment or early fibrosis, which may precede chamber enlargement. Further longitudinal studies with more sensitive imaging modalities, including global longitudinal strain (GLS), could clarify the timeline and extent of LA changes in women with prior PE [29]. GLS has emerged as a promising marker of subclinical myocardial dysfunction, capable of detecting impaired myocardial deformation even in patients with preserved ejection fraction. Despite its increasing use, GLS presents several limitations in the current assessment of left ventricular function. These include variability between different vendors and software platforms, dependence on image quality, and the lack of universally accepted reference values across diverse populations. Moreover, GLS primarily evaluates longitudinal myocardial fibers and may not fully capture global myocardial performance, especially in pathological conditions that preferentially affect circumferential or radial myocardial layers. These limitations must be acknowledged when interpreting GLS findings in both clinical and research settings. In the future, the reliability of GLS is expected to improve through the standardization of acquisition and analysis protocols, the development of vendor-independent software, and the integration of GLS data with other imaging biomarkers and clinical parameters. These advancements could enhance accuracy and prognostic utility in both research and clinical practice. The integration of advanced echocardiographic techniques into cardiovascular risk stratification could be particularly valuable in populations with subtle but cumulative risk profiles, such as women with a history of PE. Our findings underscore the critical role of echocardiography in detecting subclinical cardiovascular changes in women with a history of PE. Echocardiography remains a non-invasive, cost-effective, and highly informative tool for evaluating cardiac structure and function, particularly in high-risk populations. Its accessibility and diagnostic yield make it a cornerstone of preventive cardiology. Incorporating echocardiographic screening into postmenopausal care for women with prior PE may allow for earlier detection of cardiac abnormalities and timely initiation of preventive interventions [29]. This is especially relevant given that symptoms of heart failure or ischemic disease may be more subtle or atypical in women, leading to underdiagnosis or delayed treatment. Early detection of LVH or DD may enable clinicians to implement lifestyle changes, optimize blood pressure control, and manage metabolic comorbidities more effectively. From a clinical standpoint, our study reinforces the importance of viewing PE not only as a pregnancy complication but also as a predictor of future cardiovascular disease. Guidelines from professional societies, such as the American Heart Association, now include PE as a major risk factor in women’s cardiovascular health assessment. However, awareness remains limited, and more work is needed to translate this knowledge into routine practice. Health professionals should be encouraged to take a detailed obstetric history, including PE, when assessing cardiovascular risk in women, particularly in the peri- and postmenopausal periods [29]. Cardiovascular prevention strategies in women have historically been underutilized and under-researched. Acknowledging the role of pregnancy-related conditions like PE in shaping long-term cardiovascular health represents a step toward more individualized and effective care. By promoting a life-course approach to women’s cardiovascular risk, informed by obstetric history, we may improve both early detection and outcomes in this vulnerable population.

## 5. Conclusions

Our study demonstrates that women who experience PE are at significantly increased risk for hypertensive cardiac disease later in life, characterized by LVH and diastolic dysfunction. Furthermore, the observed familial clustering of hypertension suggests that daughters of affected mothers may also be at increased risk, highlighting the potential need for intergenerational surveillance. PE should be recognized as an early indicator of cardiovascular vulnerability, prompting lifelong monitoring and proactive risk management strategies, including lifestyle modification, pharmacologic therapy when indicated, and targeted echocardiographic evaluation. Prospective longitudinal studies are needed to confirm these associations and guide surveillance protocols.

## Figures and Tables

**Table 1:** Descriptive statistics of the entire study population.

Population Characteristics	Descriptive Statistics	
	PE	CG
All women	108	100
Years since menopause	4.7 ± 3.7	4.6 ± 3.1
Age	46.2 ± 16.2	46.6 ± 18.8
Height	161.9 ± 9	162.2 ± 8
Weight	73.2 ± 8.4	69.9 ± 9.3
BMI	28.7 ± 2.2	27.7 ± 1.8

PE—preeclampsia; CG—control group.

**Table 2:** Prevalence of cardiovascular conditions.

Cardiovascular Conditions	PE Group (*n*)	PE Group (%)	Control Group (*n*)	Control Group (%)	*p*-Value	Chi-Square	Odds Ratio
Eccentric Left Ventricular Hypertrophy	40	37	23	23	<0.02	4.8	1.9
Left Ventricular Diastolic Dysfunction	56	51	39	39	<0.003	13.2	2.6
Obesity	52	39	28	28	<0.09	2.7	1.6
Maternal History of Arterial Hypertension	59	55	26	26	<0.003	8.4	2.3
Left Atrial Enlargement	24	22	15	15	<0.1	1.8	1.6

**Table 3:** Summary of key findings and statistical significance.

Finding	PE Group (%)	Control Group (%)	Statistical Significance
Higher Prevalence of Structural and Functional Abnormalities	Significantly Higher	Lower	Yes
Eccentric Left Ventricular Hypertrophy	37%	23%	Significant (*p* < 0.02)
Left Ventricular Diastolic Dysfunction	51%	39%	Highly Significant (*p* < 0.003)
Obesity	39%	28%	Not Significant (*p* < 0.09)
Maternal History of Arterial Hypertension	55%	26%	Significant (*p* < 0.003)
Left Atrial Enlargement	22%	15%	Not Significant (*p* < 0.1)

## Data Availability

The data presented in this study are available on request from the corresponding author.
